# A systematic review and meta-analysis of *Leptospira* spp*.* infection in wildlife: Contributions to a One Health perspective

**DOI:** 10.1016/j.onehlt.2026.101433

**Published:** 2026-05-04

**Authors:** Stephanie Bergmann Esteves, Ana Carolina Monteiro Miranda Grolla, Adriana Cortez, Juliana de Paula Nhanharelli, Evelyn Moura de Lima, Felipe Fornazari, Luiz Gustavo Melo da Silva, Mariana Vitória Ramos do Amaral, Bruno Alonso Miotto

**Affiliations:** a Universidade Santo Amaro (UNISA), São Paulo, Brazil; b Faculdade de Medicina Veterinária e Zootecnia da Universidade de São Paulo, São Paulo, Brazil; c Programa de Pós-Graduação em Saúde Única (UNISA), São Paulo, Brazil; d Universidade Estadual Paulista Júlio de Mesquita Filho (UNESP), Botucatu, Brazil

**Keywords:** Leptospirosis, Surveillance, Renal shedding, Fauna, Isolation

## Abstract

Leptospirosis is a globally distributed zoonosis maintained by a wide range of mammalian hosts, yet the contribution of wildlife to pathogen circulation from a quantitative perspective remains poorly estimated. This PROSPERO-registered systematic review and meta-analysis aimed to estimate infection rates across wildlife taxa and document which *Leptospira* species, serogroups, or serovars have been detected in these hosts through direct detection of the bacterium. A systematic search of PubMed, Scopus, and Web of Science and other four databases identified studies published between 2002 and 2022 reporting direct detection of *Leptospira spp.* in wild animals. Eligible studies included observational investigations using molecular, culture-based, or immunohistochemical methods to detect the pathogen in biological samples. Data were extracted and synthesized using meta-analytical models to estimate infection prevalence and urinary shedding across taxa while descriptive analyses summarized the diversity of infecting species and serogroups. To avoid bias from synanthropic rodents, studies involving *Rattus rattus*, *Rattus norvegicus*, and *Mus musculus* were excluded from all analyses. A total of 263 studies met the inclusion criteria, comprising 54,389 individuals from 648 wildlife species. The pooled prevalence of infection was 14.5%, and renal shedding reached 16.6%, with substantial variation among taxa. Rodents and bats were the most frequently sampled hosts and exhibited the broadest diversity of *Leptospira* species and serogroups. Isolation data identified L. *interrogans*, *L. kirschneri*, and L. *borgpetersenii* as the most frequently detected species, while serogroups Icterohaemorrhagiae, Australis, and Pomona predominated among isolates. Evidence from non-mammalian taxa, although limited, suggests that reptiles and amphibians may contribute to transmission in specific ecological contexts. These findings highlight the importance of integrated wildlife surveillance and improved pathogen characterization to better understand leptospirosis ecology within a One Health framework. We advocate for improved *Leptospira* isolation protocols and expanded application of molecular tools capable of serovar-level prediction, coupled with open data-sharing platforms to enhance global collaboration.

## Highlights

• First PROSPERO-validated review on wildlife leptospirosis worldwide.
• First study to integrate molecular and serological typing.
• Meta-analysis reveals 14.5% infection and 16.6% renal shedding in wildlife.
• Rodents and bats host the widest diversity of *Leptospira* species and serogroups.

## 1 Introduction

Leptospirosis is a zoonotic disease caused by pathogenic *Leptospira*, a bacterium with a worldwide distribution that can infect a broad range of mammalian hosts, including humans, domestic animals, synanthropic species, and wildlife [1]. *Leptospira* species are classified according to their genetic features, encompassing 74 genomic species [2], [3]. Pathogenic leptospires are also distinguished by the structural diversity of their outer membrane lipopolysaccharides, giving rise to over 300 known serovars, which are grouped into 24 antigenically related serogroups [4].

Inter-species transmission in most host species occurs predominantly through indirect routes, *via* contact of the skin or mucous membranes with contaminated water or mud, highlighting the critical role of environmental factors in the infection dynamics [1]. Although recent evidence suggests that pathogenic *Leptospira* may replicate in the environment [5], their long-term persistence in nature relies primarily on the infection of maintenance hosts that asymptomatically harbor the bacterium in their renal tubules and shed it into the environment *via* urine [6].

Occasionally, infection may progress to acute illness with serious and potentially fatal outcomes, affecting renal, hepatic, pulmonary, and coagulation systems, especially when the infected host is considered to be an accidental host, meaning that the infecting serovar is not adapted to long-term survival in the host's kidneys [6], [7]. Nevertheless, mild and asymptomatic forms of infection are far more frequent, and infected animals may act as reservoir for long periods. Host-serovar adaptation, although not absolute, has been widely documented in the literature, with some animal species being classically associated as maintenance hosts for specific serovars [8]. Cattle and sheep are frequent hosts of the Sejroe serogroup [9]; dogs are considered maintenance hosts for the Canicola serogroup [10]; horses are primarily associated with the Bratislava serogroup [11]; pigs commonly harbor the Pomona serogroup [12]; and synanthropic rodents are known reservoirs of the Icterohaemorrhagiae serogroup [13].

While the role of domestic and synanthropic animals as reservoir hosts is relatively well characterized, host-serovar associations as well as the involvement of wildlife species in the transmission cycle remain poorly understood. This knowledge gap stems not only from the historical underrepresentation of wildlife in epidemiological studies of leptospirosis, but also from the tendency to treat wildlife as a single, homogeneous group. In reality, wildlife comprises an exceptionally diverse array of taxa with broad and heterogeneous geographic distribution, which precludes generalizations. To date, only a limited number of serovars are suspected to be maintained by one or a few wildlife species, whereas others – such as Grippotyphosa and Pomona – appear to be adapted to a wider range of animal hosts [14].

Yet, leptospirosis in wildlife has gained increasing attention in recent decades due to its implications for animal health, public health, and biodiversity conservation. Overall, pathogenic *Leptospira* are widely known for infecting diverse mammal species, being able to survive in regions with different environmental features [4], [15]. *Leptospira* infection has been described in many unconventional hosts, including poikilothermic animals like reptiles and amphibians [8], as well as in strict marine mammals like whales and dolphins [16]. These factors make *Leptospira* a ubiquitous pathogen, with a broad range of reservoirs and complex epidemiological scenarios that vary greatly depending on the geographical region.

Since indirect transmission is predominant, connections between wildlife and humans are largely shaped by their coexistence and shared use of environmental landscapes [17]. These interfaces are frequently intensified by abrupt land-use changes and deforestation, which foster opportunities for cross-species transmission and generate new ecological niches for the emergence, persistence, and evolution of many pathogens, including *Leptospira* [18]. This is especially true given that over 70% of emerging zoonotic diseases originate from wildlife [19], and that the intensification of human–wildlife interactions has substantially elevated the risk of pathogen spillover to human populations over the past decades [17], [19], [20]. For this reason, strengthening scientific efforts to elucidate the role of wildlife in leptospiral transmission should be considered a research priority and permanently encouraged.

Most studies addressing *Leptospira* infection in wildlife have relied on serological approaches, notably the microscopic agglutination test to assess infection. These studies typically report a high proportion of seroreactive animals, suggesting, at very least, that environmental contamination is widespread. As the MAT is only serogroup specific, and paradoxical reactions are frequent, serological data typically diverge the leptospiral strains that are actually isolated from biological samples taken from wildlife populations [15], [21]. Such incompatibility highlights the limitations of using the MAT to fully assess the epidemiology of leptospirosis in highly complex environments, with intense interactions among susceptible hosts, as seen in wildlife.

Isolation followed by molecular and serological typing is generally required to achieve identification at the serovar or serogroup level [6]. However, culturing *Leptospira* remains a major challenge due to the bacterium's fastidious growth requirements and the frequent contamination of cultures by other microorganisms. Successful isolation typically depends on the use of fresh clinical samples, which further complicates the process under field conditions, particularly during active surveillance efforts targeting wildlife. In such contexts, molecular approaches may offer important advantages, not only for diagnostic purposes, as commonly assessed by a myriad of single-gene amplification approaches [1], but also for strain characterization. DNA-based typing methods such as pulsed-field gel electrophoresis (PFGE), multilocus sequence typing (MLST), multiple-locus variable-number tandem-repeat analysis (MLVA), and whole-genome sequencing (WGS) may, in some cases, provide serovar or serogroup-level resolution, even in the absence of bacterial isolates [22].

In this context, systematic reviews incorporating molecular and microbiological data play a critical role in consolidating dispersed and methodologically heterogeneous data, identifying gaps in host-pathogen associations, and guiding future research efforts and surveillance strategies. A clearer understanding of which *Leptospira* strains are circulating in wildlife – and in which host species – can improve risk assessments of zoonotic transmission, support One Health initiatives, and contribute to evidence-based public health planning and conservation policies [23].

Early efforts in the 1960s and 1970s sought to systematize the identification of leptospiral strains and geographic distribution of infected mammalian hosts [24], [25]. Since then, the literature has expanded considerably, underscoring the need for periodic reviews that offer a comprehensive synthesis of the available data. Fratini et al. [26] and Cilia et al. [8] provided an extensive overview of leptospirosis in wildlife, highlighting the involvement of a wide range of animal hosts in the transmission cycle. Similarly, Vieira et al. [27] and Guernier et al. [28] have also provided important contributions; however, these studies were either geographically restricted or not designed as systematic reviews. Other reviews have focused narrowly on specific taxonomic groups, such as bats [29], [30], [31], [32], non-human primates [33], marine mammals [16], and rodents [13], leaving a gap in comprehensive assessments that consider the full breadth of wildlife taxa.

More recently, two systematic reviews conducted by Browne et al. [34] and Hagedoorn et al. [14] have addressed the identification and distribution of *Leptospira* serogroups and serovars in animal hosts. While these works represent significant progress, their broader scope – including domestic and synanthropic animals – may have resulted in limited emphasis on wildlife-specific data. Additionally, molecular data were not incorporated, limiting the inclusion of studies that relied on DNA-based methods for *Leptospira* detection and typing, particularly those in which bacterial isolation was unsuccessful.

In light of these potential gaps, there remains a need for a systematic and wildlife-centered synthesis of the available literature, including multiple approaches for proper leptospiral identification. To address this, the present study undertakes a systematic review with meta-analysis specifically focused on leptospirosis in wild animals, with the aim of estimating infection positivity rates across different wildlife taxa and identifying which *Leptospira* species, serogroups, or serovars have been reported in these hosts through direct bacterial detection methods. We hope that this effort will help consolidate existing evidence, identify critical knowledge gaps, and offer guidance for future research and surveillance strategies targeting wildlife reservoirs of *Leptospira* spp..

## 2 Methods

This systematic review was conducted in accordance with the Preferred Reporting Items for Systematic Reviews and Meta-Analyses 2022 guidelines (PRISMA), with additional validation and registration on the International Prospective Register of Systematic Reviews (PROSPERO - protocol CRD42021259993).

### 2.1 Eligibility criteria

Full-text articles (observational studies or case reports) describing the direct detection of *Leptospira spp.* in wild or free-ranging animals were considered for selection. Detailed eligibility criteria, including the description of species that were considered as wildlife, can be found in the Supplementary Table 1. Studies focusing exclusively on domestic animals, humans, and synanthropic fauna were excluded. Studies evaluating multiple species, including these groups, were retained during the selection stage for further assessment. In such cases, appropriate precautions were taken during the analyses to ensure that data from these species were excluded. Accordingly, *Rattus rattus*, *Rattus norvegicus*, and *Mus musculus* were removed from all analyses.

Direct detection methods encompassed bacterial culture, PCR, other molecular techniques, and optical microscopy. Eligible studies had to be published between January 2002 and April 2022, and written in English, Spanish, or Portuguese, regardless of country of origin. Studies based solely on indirect detection methods (mostly MAT) or involving *in vivo* experimental infections were excluded. Studies reporting only negative molecular detection results were not excluded to avoid bias in the positivity rate estimates. Unpublished studies, conference abstracts, dissertations, literature reviews, systematic reviews, and experimental studies were excluded, as well as studies lacking a quantitative description of the results or not available through digital databases.

### 2.2 Information sources and search strategy

The literature search was carried out in seven electronic databases, including PubMed, SciELO, Embase, Web of Science, BVS (Virtual Health Library), Scopus, and LILACS. The search terms included: (leptosp* OR “l. biflexa” OR “l. interrogans” OR “l. borgpetersenii” OR “l. kirschneri” OR “l. licerasiae” OR “l. santarosai” OR “l. weilii”) AND (wild OR wildlife OR nondomestic OR reserve OR fauna OR zoo OR mammal? OR carnivore? OR bird? OR reptile? OR amphibian? OR cetacean? OR colonial bird? OR seabird? OR primate? OR marsupial? OR chelonian?) AND (isolated OR isolation OR isolates OR culture OR molecular OR DNA OR urinalysis OR detection OR PCR OR sequenc* OR characterization OR identification). Slight adaptations in the search strategy were carried out according to each database used (Supplementary Table 2).

An initial search was performed on April 1, 2022, and all retrieved entries were exported to reference management software for subsequent screening and selection. Additionally, a snowballing strategy was employed by examining the reference lists of all included studies to identify potentially relevant articles not captured in the database searches. An additional snowball search was carried out on May 05, 2025 and included the most prominent reviews addressing *Leptospira* infection in wildlife [8], [12], [13], [14], [26], [27], [28], [29], [30], [33], [34], [35], [36], [37].

### 2.3 Selection and data extraction process

After retrieval, duplicate entries were removed using Mendeley Reference Management software. The titles and abstracts of all identified articles were screened and categorized as either “excluded” or “potentially eligible”. Studies classified as “potentially eligible” were retrieved in full and assessed by two independent reviewers (AG and EM) according to the predefined eligibility criteria. Discrepancies were resolved through discussion with a third reviewer (SBE). For the snowball search, one author (BAM) screened the reference lists of the included articles, and another author (SBE) verified eligibility according to the predefined eligibility criteria.

Data extraction was performed independently by two reviewers (AG and EM) using a standardized spreadsheet after prior training. Discrepancies in data extraction were reviewed and resolved by three senior authors (BAM, AC, and SBE).

### 2.4 Data items

Data from the studies were extracted to a standardized spreadsheet and organized into three separate worksheets:

I. General study-level data: Information collected at the study level included: first author, year of publication, country, continent, sampling period, number of animal species evaluated, total sample size, animal setting (free-ranging, captive, or both), whether sample size calculation was reported, sampling method, and types of diagnostic tests conducted. The tests were recorded in a binary format (yes/no) for the following categories: bacterial culture and isolation, molecular detection, immunohistochemistry (IHC), serogroup or species identification, and renal shedding testing.
II. Species-level data: For each animal species evaluated within the study, the following information was extracted: class, family, and species name; total number of individuals tested and the number testing positive for each direct detection method; type of sample collected (*e.g.*, kidney, blood, liver); *Leptospira* species identified; and whether any discrepancies were observed between test results (*e.g.*, positive in one test but negative in another).
III. Isolate-level data: For each *Leptospira* isolate, we recorded: the host animal information (species, class, family, and sample origin), isolate identification (ID), whether molecular characterization was performed, the target gene(s), and the method used (*e.g.*, VNTR, MLVA, MLST, RAPD, PFGE, or WGS). If serological identification was performed, we noted the technique used (*e.g.*, polyclonal or monoclonal antibodies, MAT, or CAAT), as well as the identified *Leptospira* species, serogroup and serovar.

### 2.5 Risk of bias assessment

The risk of bias for each included study was assessed using an adapted version of the Joanna Briggs Institute (JBI) critical appraisal checklists for observational studies, based on the JBI Manual for Evidence Synthesis [38]. For each article, the percentage of items rated as “Yes,” “No,” or “Unclear” was calculated to provide an overall qualitative assessment of study reliability in relation to the review question.

Seven domains were evaluated for each article and rated as “*Yes*”, “*No*” or “*Unclear*” based on predefined criteria. The first domain assessed whether the inclusion criteria were clearly defined. Studies were considered adequate if the authors provided clear information regarding animal selection, such as clinical condition (*e.g.*, symptomatic, asymptomatic, vaccinated), geographic origin, and justification for inclusion. In the second domain, we evaluated whether the study subjects and setting were described in sufficient detail. This included a clear description of the animal population, including the description and identification of the species sampled, with species-level data, sampling location, and collection period. Studies lacking species-specific information or presenting aggregated data without differentiation were considered unclear. The third domain examined the validity and reliability of exposure measurement. For culture-based methods, we assessed whether the study reported the time between sample collection and inoculation, as well as storage conditions. For PCR-based detection, particular attention was paid to the handling of urine samples, considering prolonged freezing may affect DNA integrity. The fourth domain investigated whether standardized and objective criteria were used to measure the condition. For culture, studies that relied solely on visual indicators (*e.g.*, turbidity, ring formation, dark-field microscopy) without molecular or serological confirmation of isolates were considered at high risk. For molecular tests, we verified whether a consistent protocol was applied across all samples and species. The fifth and sixth domains addressed the identification and handling of potential confounding factors. We considered whether the authors used previously validated PCR primers or developed and validated new ones, and whether adjustments or controls for these variables were discussed. Finally, the seventh domain evaluated the validity and reliability of the results. Studies that reported concordant results across culture and PCR, or that confirmed PCR positivity through sequencing or additional methods, were rated positively. Studies with PCR alone, without sequencing, were considered at higher risk of bias. Studies lacking such description were rated as having “unclear” potential bias.

Potential risk of bias across studies, such as publication bias or small-study effects, was explored through visual inspection of funnel plots. When appropriate, asymmetry was further assessed using Egger's regression test.

### 2.6 Data synthesis and statistical analysis

A critical, qualitative analysis was conducted to examine the main characteristics, methodological aspects, and limitations of the included studies. Descriptive statistics were used to summarize the extracted data. For categorical variables, results were expressed as absolute and relative frequencies, along with 95% confidence intervals (CI) calculated using the Clopper–Pearson exact method. For continuous variables, the analysis included measures of central tendency and dispersion, such as mean, standard deviation, median, interquartile range (IQR), and range.

Where sufficient and comparable data were available, pooled positivity rates for the direct detection of *Leptospira* spp. were estimated through meta-analysis models using the inverse variance method [39]. Proportions were primarily pooled using raw proportion models (PRAW). When datasets contained a high number of zero events, a logit transformation (PLOGIT) was applied to stabilize variance before pooling. The polled estimates were presented using forest plots, and heterogeneity among studies was assessed using the I^2^ statistic [40]. Subgroup analyses were conducted to explore potential sources of heterogeneity according to geographic region (continent), taxonomic class, family, and type of diagnostic test used (*e.g.*, culture, PCR, IHC).

When significant heterogeneity was identified (I^2^ > 50%), random-effects models were applied; otherwise, fixed-effects models were used. Pooled estimates for positivity rates were reported with 95% CI. All analyses were performed in the R statistical environment (R version 4.4.2) using the “*meta*” package (version 8.0–1) for meta-analysis [40]. The R codes used for the analyses can be made available upon request.

## 3 Results

### 3.1 Study selection

The systematic search yielded a total of 6581 entries, which were recovered from the following databases: PubMed (*n* = 3205), Web of Science (*n* = 1281), Scopus (*n* = 765), EMBASE (*n* = 715), LILACS (*n* = 589), and SciELO (*n* = 26). After removing 2493 duplicates, 4088 articles remained for title and abstract screening. Of these, 3269 and 499 articles were excluded based on title and abstract screening, respectively, resulting in 320 studies selected for full-text review. Four articles were not available digitally, and the remaining 316 full-text articles were assessed for eligibility.

**Fig. 1:**
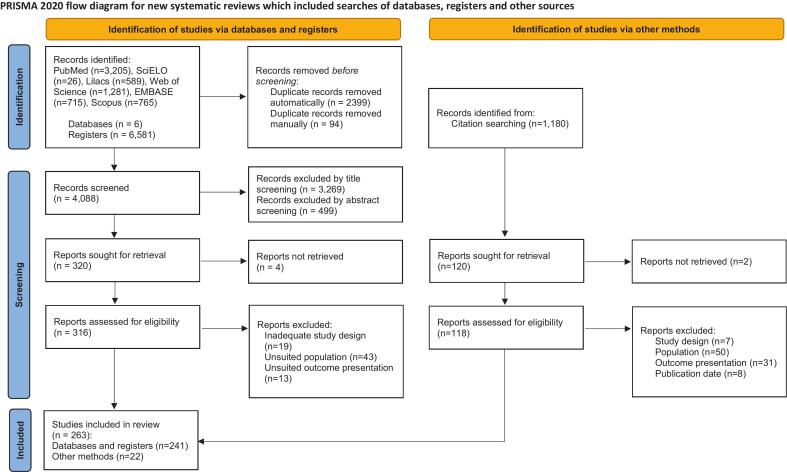
PRISMA flowchart showing the selection of studies eligible for this systematic review.

Following full-text assessment, 75 articles were excluded due to: inadequate study design (*n* = 19), unsuitable study population (*n* = 43), or outcome reporting not aligned with inclusion criteria (*n* = 13). An additional 1180 articles were identified through citation searching (snowballing strategy). In total, 263 studies met the eligibility criteria and were included in this review, with 241 studies identified through database and register searches, and 22 retrieved through citation searching. Fig. 1 shows a summary of the selection process, including the reasons that led to the exclusion of articles during the full-text reading stage.

### 3.2 Study characteristics

**Fig. 2:**
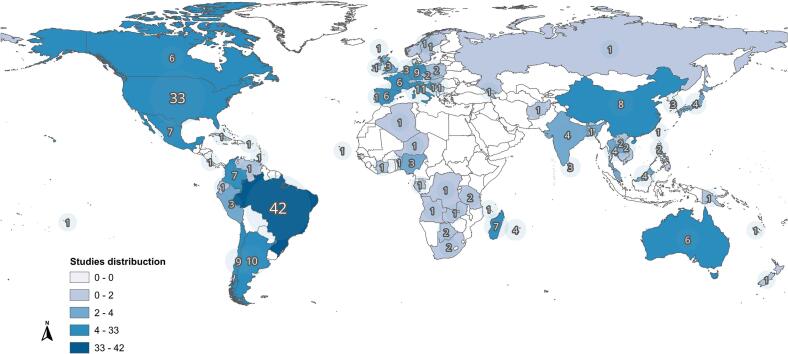
Global distribution of studies included in this systematic review.

Given the large volume of information extracted from the selected studies, the data are presented in their raw form to facilitate objective consultation (Supplementary Table 3). The full list of references can also be found in Supplementary Table 4. Among the 263 articles included, 231 (87.8%) were eligible for the overall analysis of infection frequency in wild animals, and 57 (21.7%) provided data on isolates recovered from wild species. The Americas represented the continent with the highest number of studies, accounting for 123 articles (46.8%), of which 27.8% were from South America, 17.1% from North America, 1.9% from Central America (Fig. 2). Europe accounted for 60 articles (22.8%), followed by Asia with 36 (13.7%), Africa with 34 (12.9%), and Oceania with 10 (3.8%). The countries with the highest number of studies were Brazil (42 studies; 16.0%), USA (32; 12.2%), and Italy (11; 4.18%). A full list of countries and detailed characteristics of the studies included can be found in Supplementary Table 3.

Regarding the study populations, most articles focused on wild-caught animals (227 studies; 87.3%), while 18 (6.9%) involved captive populations, and 15 (5.8%) included both types of animals. Among studies on captive animals, 50% were conducted in zoos and 50% in other kinds of managed environments. Only 3 out of the 263 studies (1.1%) reported sample size calculation, and just one study employed a probabilistic sampling method. The number of animals included in each study ranged from 1 to 3950, with a mean of 256 animals (SD ± 536). Additionally, the number of species analyzed per study ranged from 1 to 69, with a mean of 5 species per study (SD ± 7).

### 3.3 Risk of bias in studies

The risk of bias assessment was performed for all studies included. In the first domain, only 10.3% of studies were rated “*Yes*”, while 6.5% were rated “*No*” and the majority (83.3%) were classified as “*Unclear*,” often due to insufficient description of the characteristics used to select study populations. For the second domain, 69.6% of studies were rated “*Yes*”, 18.3% as “*Unclear*” and 12.2% as “*No*”. Most studies that failed to meet this criterion lacked species-level data or did not differentiate results by animal origin or sampling site. For the third domain, 69.4% of studies were rated positively, indicating adequate reporting of sample handling procedures, especially for molecular and culture-based methods. However, 20.5% were classified as “*Unclear*” and 10.1% as “*N*o” mainly due to lack of methodological detail for critical steps such as DNA extraction from urine or sample preservation for culture. The fourth domain was the best-performing domain, with 93.1% of studies rated as “Y*es*”, 5.0% as “*Unclear*” and only 1.5% as “No”. Domains five and six jointly evaluated the identification and management of potential confounders, such as primer validation or consistency in diagnostic protocols across species. These domains had 83.3% of studies rated as “*Yes*”, 12.2% as “*No*” and 4.6% as “*Unclear*”. Finally, the seventh domain revealed slightly more variability. While 66.2% of studies were rated “*Yes*” 20.9% were classified as “*No*” and 12.9% as “*Unclear*”. Studies that relied solely on PCR without sequencing or supporting confirmatory methods were more likely to be at higher risk of bias in this domain.

When analyzing the studies individually, the majority showed moderate to low risk of bias, with a median of 65.3% of domains rated “*Yes*” per article. The full study-level evaluation is available in Supplementary Table 4.

### 3.4 Direct detection of *Leptospira*

Among the 263 studies that performed direct detection of *Leptospira spp.*, data were disaggregated by host species, resulting in 1083 reports and 54,389 individuals. Altogether, *Leptospira* detection was assessed in 648 wild species, distributed across 7 taxonomic classes, 30 orders and 92 families. Mammals accounted for the vast majority of reports (1030; 95.1%; 53,529 individuals), followed by reptiles (32 reports; 2.9%; 541 individuals), amphibians (14 reports; 1.3%; 267 individuals), birds (2 reports; 0.2%; 44 individuals), and protostomes (2 reports of gastropods, 0.2%, 2 individuals; 1 report of insects, 0.1%, 2 individuals; and 1 report of malacostraca, 0.1%, 1 individual).

The three most frequently sampled animal orders were Rodentia (410 individual reports; 37.9%; 31,685 individuals), Chiroptera (234 reports; 21.6%; 5415 individuals), and Carnivora (130; 12.0%; 6563 individuals). Other mammalian orders were also represented, although at lower proportions, such as Eulipotyphla (*e.g.,* hedgehogs, moles and shrews - 54 reports; 5.0%; 2081 individuals), Didelphimorphia (*e.g.,* opossums) 48 reports; 4.4%; 539 individuals), Afrosoricida (*e.g.,* golden moles and tenrecs - 35 reports; 3.2%; 630 individuals), Artiodactyla (*e.g.,* antelopes, gazelles, suids and deer - 34 reports; 3.1%; 4927 individuals), and Primates (31 reports; 2.9%; 640 individuals). In contrast, non-mammalian orders appeared with lower frequencies, including Squamata (*e.g.,* phytons, vipers - 19 reports; 1.8%; 219 individuals), Testudines (*e.g.,* turtles - 11 reports; 1.0%; 254 individuals), Anura (*e.g.*, frogs and toads - 7 reports; 0.7%; 189 individuals), and Caudata (*e.g.,* salamanders - 7 reports; 0.7%; 78 individuals), as well as birds (Charadriiformes *e.g.,* seagulls - *n* = 43 individuals; and Galliformes *e.g.*, partridges - *n* = 1), and invertebrates (Coleoptera *e.g.,* beetles *n* = 2, Decapoda - shrimps n = 1, and Stylommatophora *e.g.,* snails n = 2).

Molecular detection was carried out in 208 articles (79.1%), totaling 924 reports (85.3%; 95% CI: 83.1–87.4%). Sample types included blood (89 reports; 1913 individuals tested, 174 positives), urine (85 reports; 2604 individuals tested, 365 positives), kidney (662 reports; 34,968 individuals tested, 4135 positives), and other sample types (164 reports; 3932 individuals tested, 676 positives), which included abscesses, milk, feces, liver, spleen, uterine fluid, lymph nodes, lungs, placenta, fetal tissues, nervous system, and reproductive organs (Table 1).

**Table 1:** Molecular detection and species identification of *Leptospira* by sample type.

Sample type	N of reports	Individuals tested	Total positives	% positive (95% CI)	Identified species (n)
Blood	89	1913	174	9.10% (7.84–10.48%)	*L. interrogans* (50)
Urine	85	2604	365	14.02% (12.68–15.45%)	*L. interrogans* (51)*, L. borgpetersenii* (38)*, L. santarosai* (9)*, L. kirschneri* (6)
Kidney	662	34,968	4135	11.83% (11.48–12.19%)	*L. interrogans* (635)*, L. kirschneri* (627)*, L. borgpetersenii* (220)*, L. noguchii* (24)*, L. weilii* (22)*, L. kmetyi* (20)*, L. mayottensis* (10)*, L. fainei* (9)*, L. biflexa* (6)*, L. santarosai* (8)*, L. meyeri* (3)
Others	164	3932	676	17.19% (15.97–18.47%)	*L. interrogans* (57)*, L. borgpetersenii* (48)*, L. kirschneri* (20)*, L. mayottensis* (8)*, L. fainei* (6)*, L. wolffii* (2)

Note: The same study or individual may appear for more than one sample type.

Culture and isolation of *Leptospira* were performed in 342 reports (104 articles), corresponding to 31.6% of all reports involving wild animals (95% CI: 28.8%–34.3%). The kidney was the tissue most frequently tested, accounting for 266 reports, in which 11,501 animals were tested and 598 presented positive results. Urine samples were used in 44 reports, with 702 animals tested and 58 positives, while blood samples were analyzed in 15 reports, yielding 16 positives out of 221 animals tested. Other types of samples — including abscesses, milk, liver, spleen, uterine fluid, lungs, and mixed organ pools — were used in 33 reports, testing 3000 animals, with 70 positive cases.

A total of 808 leptospiral isolates with some degree of characterization was described. Most of these originated from Asia (51.1%), followed by Europe (29.1%), South America (8.4%), North America (7.6%), Africa (3.6%), and Central America (0.3%). The isolates were obtained from 87 different wild animal species, distributed across four vertebrate classes, 15 orders and 34 taxonomic families. Mammals were overwhelmingly predominant, representing 99.5% (804 isolates), while amphibians, birds, and reptiles contributed with just one or two isolates each (≤0.25% each). Among the 808 isolates, only 732 had available data regarding the type of sample used for culture and isolation. Regarding these samples, the urinary tract was the primary source, accounting for 96.9% of characterized isolates (710/732; 95% CI: 94.8%–97.6%). A smaller proportion of isolates were derived from blood (2.2%; 95% CI: 1.1%–3.3%), and from reproductive organs (0.82%; 95% CI: 0.17%–1.47).

The IHC detection was described in 22 articles (8.4%), corresponding to 32 reports that involved kidney samples, with 955 animals tested and 195 testing positive (20.4%). The remaining four reports evaluated liver samples, testing 21 animals, of which 3 were positive (14.3%).

The frequency of *Leptospira* detection was further explored using multiple meta-analysis models, including sub-group analysis according to different taxonomic classifications (class, order, family), type of tests and type of sample (Supplementary Table 5). A total of 228 studies provided animal species-level data suitable for inclusion, resulting in 1252 individual records. The overall pooled prevalence of *Leptospira* infection across all vertebrate hosts was 14.5% (95% CI: 13.2–15.7%), with high heterogeneity among studies (I^2^ = 87.3%, τ^2^ = 0.0322, *p* < 0.001).

The diagnostic methods used also influenced the pooled prevalence estimates. Animals tested by molecular techniques yielded a pooled prevalence of 16.3% (95% CI: 14.8–17.7%) with high between-study heterogeneity (I^2^ = 88.4%). Culture and isolation showed a markedly lower pooled prevalence (5.7%; 95% CI: 4.4–7.0%), while immunohistochemistry (IHC) was associated with a higher overall prevalence (34.3%; 95% CI: 20.5–48.0%). The magnitude of heterogeneity remained consistently high across methods, suggesting that other variables may influence the differences in the pooled prevalences found.

**Fig. 3:**
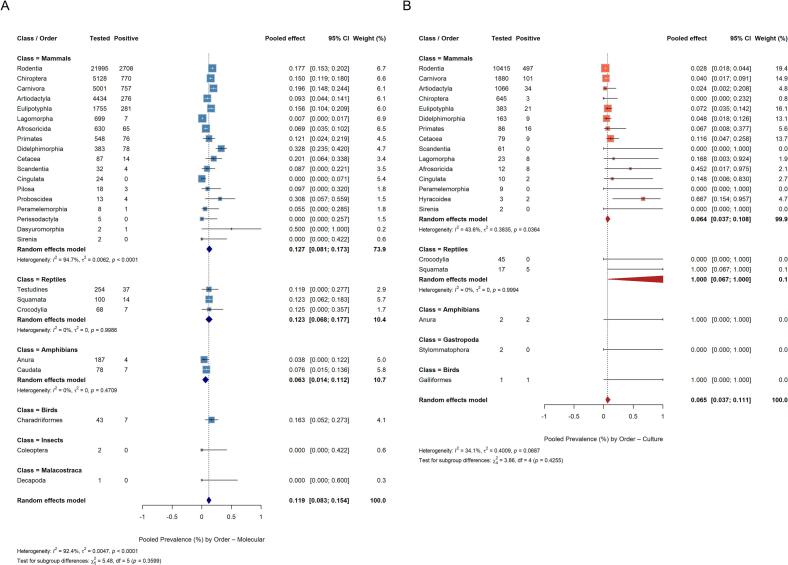
Pooled prevalence for molecular detection of *Leptospira spp.* by taxa (A) and pooled prevalence for culture of *Leptospira spp.* by taxa (B).

Considering exclusively the population in which molecular detection was carried out, which represented the largest quantity of data (*n* = 41,497 individuals), heterogeneity was particularly evident within mammalian orders (Fig. 3A). Didelphimorphia presented higher pooled prevalence (32.8%; 95% CI: 23.5–42.0%), followed by other animal orders with significant sample sizes, such as Carnivora with 19.6% (95% CI: 14.8–24.4%), Rodentia with 17.7% (95% CI: 15.3–20.2%), Eulipotyphla with 16.8% (95% CI: 10.4–20.9%) and Chiroptera with 14.9% (95% CI: 11.8–18.0%). Cetaceans (20.1%; 95% CI: 6.4–33.8%) were also noteworthy despite smaller sample sizes. Artiodactyls (9.3%; 95% CI: 4.4–14.1%) and lagomorphs (*e.g.,* rabbits - 0.7%; 95% CI: 0.0–1.7%), on the other hand, showed considerably lower values.

Family-level estimates confirmed substantial variability (Supplementary Table 5). Cricetidae (*e.g.,* hamsters, voles and muskrats - *n* = 12,587) had a prevalence of 23.9% (95% CI: 18.9–28.8%), Muridae (*e.g.,* wild mice, rats and gerbils - *n* = 7334) 15.6% (95% CI: 11.8–19.3%), and Didelphidae (*n* = 383) 32.8% (95% CI: 23.5–42.0%). Several bat families showed moderate prevalence, including Phyllostomidae (10.3%) and Molossidae (10.3%), while Vespertilionidae reached 23.9%. Some carnivore families, such as Mustelidae (16.2%) and Canidae (17.1%), also demonstrated substantial infection rates. In contrast, Bovidae (*n* = 72), Cervidae (*n* = 1226), and Podocnemididae (*e.g.,* freshwater turtles - *n* = 109) reported no positive cases despite considerable sample sizes.

Considering methodological factors, sample type showed a strong influence on the pooled prevalence. Direct detection on renal tissue or urine samples, the predominant material tested, showed a pooled prevalence of 16.5% (95% CI: 14.9–18.2%), whereas blood-based detection was lower (7.2%; 95% CI: 3.4–11.0%). Tissue samples resulted in 11.7% positivity, while studies using multiple or unspecified tissues reported higher prevalence (20.2%; 95% CI: 15.3–25.0%).

When restricting the analysis exclusively to studies in which culture-based isolation was conducted, the overall prevalence among vertebrates was markedly lower than that obtained with molecular methods, with a pooled estimate of 5.2% (Fig. 3B). However, results varied considerably across taxonomic groups, with some families showing higher-than-expected positivity. At the class level, mammals dominated the dataset (*n* = 14,837), with a prevalence of 5.2% (95% CI: 4.0–6.4%). Rodents were the main hosts sampled (*n* = 10,415), but prevalence was only 2.8% (95% CI: 1.8–4.4%), with Cricetidae and Muridae presenting very low pooled prevalence (2.0% and 4.4%, respectively). Similarly, bats, despite substantial representation (*n* = 645), showed almost null isolation (0.05%; 95% CI: 0.0–23.2%). In contrast, some small families showed high detection, such as Caviidae (*e.g.,* capybara, cavy, and guinea pig - 33.6%; 95% CI: 4.0–63.2%), Leporidae (*e.g.,* hares - 36.2%; 95% CI: 0.0–81.6%), and Bovidae (83.0%; 95% CI: 67.5–98.6%). Similarly, Nesomyidae (Madagascar rodents - 46.6%), Bathyergidae (*e.g.,* mole rats 50.0%), and Tenrecidae (41.9%) also exhibited high pooled prevalence, Cetacean families like Delphinidae (20.5%) also showed notable prevalence compared to terrestrial mammals.

**Fig. 4:**
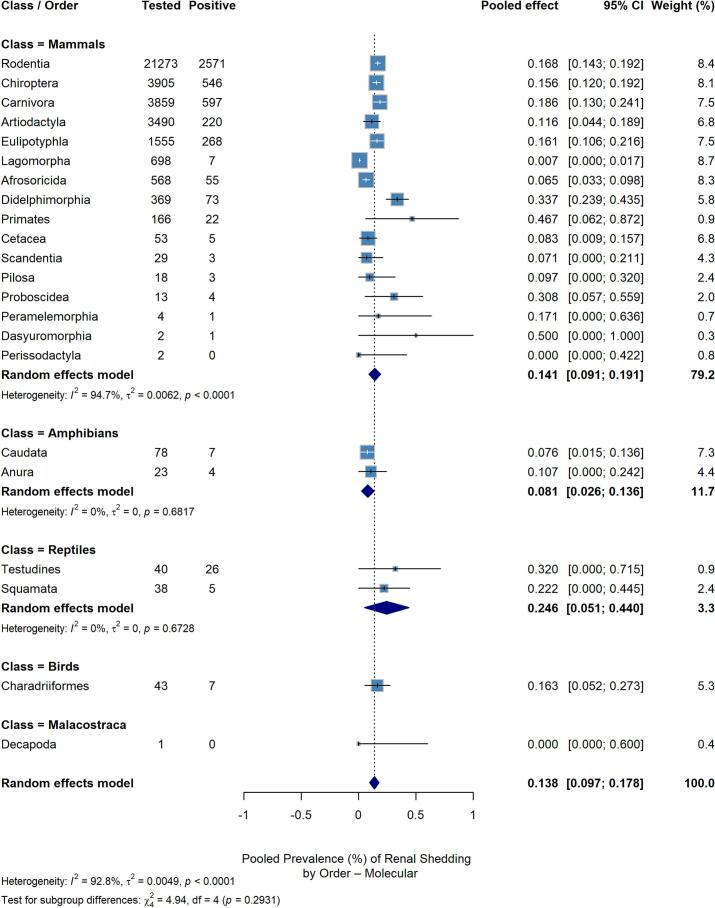
Pooled prevalence of *Leptospira spp.* detection exclusively in urine and renal samples by animal order, grouped by taxonomic class by molecular detection.

To assess the potential role of urinary shedding across different animal taxa and its implications for environmental contamination, Fig. 4 presents a pooled prevalence analysis based on molecular identification of renal shedding. Evidence of renal shedding – through urine, kidney, or urinary bladder samples – was assessed in 232 studies (88.9%). The overall pooled prevalence of *Leptospira* infection across all vertebrate hosts was 13.8%, showing similar heterogeneity patterns within mammalian orders as observed previously (Fig. 3A). Dasyuromorphia (Tasmanian devil) and Primates exhibited the highest prevalence of renal shedding (50% and 46.7%, respectively), although with notably reduced sample sizes and wide confidence intervals. Didelphimorphia showed a positivity rate of 33.7%, but the sample size was substantially smaller compared to Rodentia, Chiroptera, Eulipotyphla, and Carnivora, all of which displayed similar positivity rates consistent with the general infection rates presented in Fig. 3A.

### 3.5 *Leptospira* identification

Species-level identification through molecular approaches applied directly to biological samples was achieved in 590 of the reports (63.8% - Table 1), while the remaining focused on using molecular tools only for diagnostic purposes. The main methods used for *Leptospira* species determination were sequencing PCR products (519 reports), followed by VNTR (43), MLST (26), and MLVA (3).

Molecular characterization of isolates was performed in 731 out of the 808 samples (90.5%; 95% CI: 88.5%–92.5%), and species-level identification of *Leptospira* was achieved in 730, revealing broad taxonomic diversity among hosts (Supplementary Table 6). Conventional PCR followed by sequencing was the most common technique used (73.5%), followed by (MLST, 59.6%), multilocus variable-number tandem-repeat analysis (MLVA, 16,6%), pulsed-field gel electrophoresis (PFGE, 13,3%), variable-number tandem-repeat (VNTR, 1,9%), and whole genome sequencing (WGS, 1,4%).

A total of 519 isolates were classified as *L. interrogans*, detected predominantly in mammals (*n* = 517) and also in two amphibians from the order Anura (family *Ranidae*). Within mammals, *L. interrogans* was distributed across seven orders and 23 families, with the highest representation in Rodentia (*n* = 396; mainly *Muridae* = 365), followed by Carnivora (60; including *Mustelidae* = 16 and *Procyonidae* = 15), Primates (22; mainly *Cebidae* = 20), and Eulipotyphla (16; *Erinaceidae* = 14). Other orders, such as Artiodactyla, Cetacea, Chiroptera, Cingulata (*e.g.,* armadillos), Lagomorpha, and Scandentia (*e.g.,* tree shrews), were also represented, though in lower numbers.

*L. borgpetersenii* was identified in 129 mammal species, spanning five orders. Most isolates were from Rodentia (85; mainly *Muridae* = 78), followed by Carnivora (28; especially *Herpestidae* = 25), Eulipotyphla (10), Didelphimorphia (5), and a single isolate in Artiodactyla (*Suidae*).

*L. kirschneri* (*n* = 62) was also exclusive to mammals, occurring across four orders. The majority were in Rodentia (50; with *Muridae* = 48), followed by Carnivora (9; mostly *Procyonidae* = 8), and isolated cases in Artiodactyla (*Suidae* = 2) and Chiroptera (*Molossidae* = 1). Other species were more restricted: *L. mayottensis* (*n* = 11) occurred only in Afrosoricida (*Tenrecidae*), *L. santarosai* (*n* = 5) in Rodentia (*Caviidae*), and *L. alstonii* (*n* = 3) in Eulipotyphla (*Soricidae*). In addition, one isolate was identified as *L. interrogans* or *L. johnsonii* (*Mustelidae*), and another as *L. weilii* (*Muridae*).

**Fig. 5:**
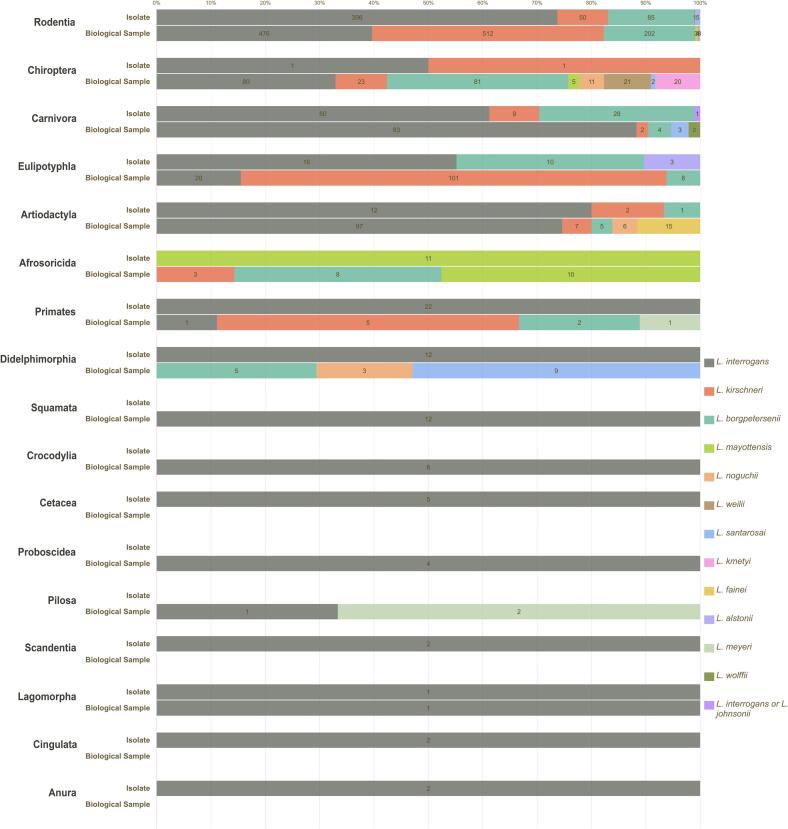
*Leptospira* species identified by molecular methods, based on both isolated strains and direct analysis of biological samples, according to host taxa.

Fig. 5 illustrates a graphical representation of *Leptospira* species identified by molecular methods, based on both isolated strains and direct analysis of biological samples, and organized according to host taxa.

From a perspective that considers different animal taxa rather than *Leptospira* species, the combined analysis (isolation plus molecular data) presented in Fig. 5 reveals that Chiroptera is infected by the widest range of *Leptospira*, with eight genomic species identified. Most of these records were obtained through molecular detection in biological samples. This analysis also revealed that Rodentia is associated with additional *Leptospira* species beyond those identified through isolation alone, including *L. mayottensis* and *L. noguchii*. Still, *L. interrogans*, *L. kirschneri*, and *L. borgpetersenii* were the predominant species found among rodents. The predominance of these *Leptospira* species was also detected in Carnivora, Artiodactyla, Eulipotyphla, and Primata, although Carnivora and Artiodactyla showed evidence of a broader diversity of infecting species. Notably, Afrosoricida was predominantly infected by *L. mayottensis*. The remaining animal orders were infected mostly by *L. interrogans*.

Regarding serogroup identification, 753 isolates (77.0%; 95% CI: 74.2–79.9%) underwent serological characterization, mainly through MAT with polyclonal antisera (*n* = 596), while monoclonal antibodies were applied in 44 (Supplementary Table 6). The most frequent serogroup was Icterohaemorrhagiae (*n* = 305), detected across a wide host spectrum. It was found predominantly in Rodentia (*n* = 268; especially Muridae = 244), but also in Primates (22; mainly Cebidae = 20), Carnivora (10), and several other mammalian orders. Interestingly, a single isolate was reported in a bird (*Phasianidae*).

The second most frequent serogroup found was Australis (*n* = 145), almost entirely restricted to mammals (*n* = 144), with emphasis on Rodentia (103; Muridae = 84), but also present in Carnivora (16), Eulipotyphla (15), and Artiodactyla (10). A single isolate occurred in an amphibian (*Ranidae*).

Pomona (*n* = 86) was identified in mammals from diverse orders, especially Rodentia (54; Muridae = 52), but also in Artiodactyla (13) and Carnivora (15), in addition to a few isolates in Cetacea and Cingulata. Javanica (*n* = 71) was mostly confined to Rodentia (66; Muridae = 65), with a small proportion in Eulipotyphla (5).

Less frequent but notable serogroups included Sejroe (*n* = 38), concentrated in Rodentia (12) and Carnivora (25; Herpestidae); Grippotyphosa (*n* = 32), broadly distributed across Rodentia (25), Artiodactyla (3), Carnivora (2), and even in a reptile (Pythonidae); Autumnalis (*n* = 30), almost exclusively in Rodentia (29; Muridae); and Hebdomadis (*n* = 15), with records in Rodentia (5), Carnivora (6), and Artiodactyla (3). Other serogroups were rare, with fewer than 15 isolates each, such as Ballum (14), Bataviae (5), Hebdomadis/Sejroe (4), Canicola (3), Pyrogenes (2), and single isolates belonging to serogroups Hardjo, Shermani, and Tarassovi.

**Fig. 6:**
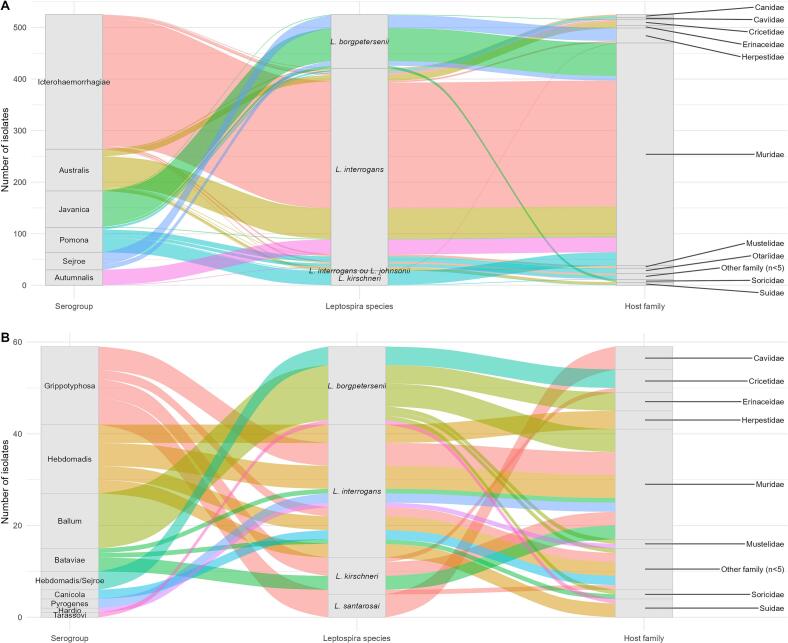
Sankey diagrams showing the relationship between *Leptospira* serogroups, *Leptospira* species, and host families based on the number of urinary isolates. The width of each flow is proportional to the number of isolates linking each category. To improve visualization, the data were divided into two panels according to the total number of isolates per serogroup. (A) Serogroups with ≥20 isolates. (B) Serogroups with <20 isolates.

Fig. 6 includes 584 isolates (77.6% out of the total 753 isolates) that were obtained from urine samples and included simultaneous molecular identification of *Leptospira* species and serogroup identification by serological approaches, allowing further insights into host-serogroup specificity relations.

Most isolates were assigned to *L. interrogans*, particularly within the serogroups Icterohaemorrhagiae, Australis, Autumnalis, and Pomona. Icterohaemorrhagiae predominated in Rodentia, especially Muridae (*n* = 244), with additional occurrences in Carnivora (Canidae, Herpestidae, Mustelidae) and other rodent families. Australis and Autumnalis were also mainly associated with *L. interrogans* in Rodentia (notably Muridae), with Australis additionally detected in Eulipotyphla, Carnivora, and Artiodactyla. The Javanica serogroup was primarily linked to *L. borgpetersenii* in Rodentia (Muridae, *n* = 64). Pomona showed a broader host range across Carnivora, Rodentia, and Artiodactyla and was associated with both *L. interrogans* and *L. kirschneri*. Sejroe was exclusively linked to *L. borgpetersenii*, mainly in Carnivora (Herpestidae) and Rodentia (Muridae). Less frequent serogroups (*n* < 20) exhibited more heterogeneous distributions across *Leptospira* species and host taxa, predominantly in Rodentia but also in Eulipotyphla, Carnivora, and Artiodactyla.

When considering the data by host taxa rather than *Leptospira* species or serogroups, Rodentia was found to shed the widest variety of *Leptospira* (12 serogroups), followed by Carnivora (7 serogroups), Eulipotyphla (5 serogroups) and Artiodactyla (4 serogroups). On the other hand, few families within the order Carnivora appear to be associated with one or few serovars. Herpestidae seem to be associated with Sejroe, Mephitidae with Grippotyphosa, and Otariidae and Phocidae with Pomona.

### 3.6 Risk of bias across studies

**Supplementary Fig. 1:**
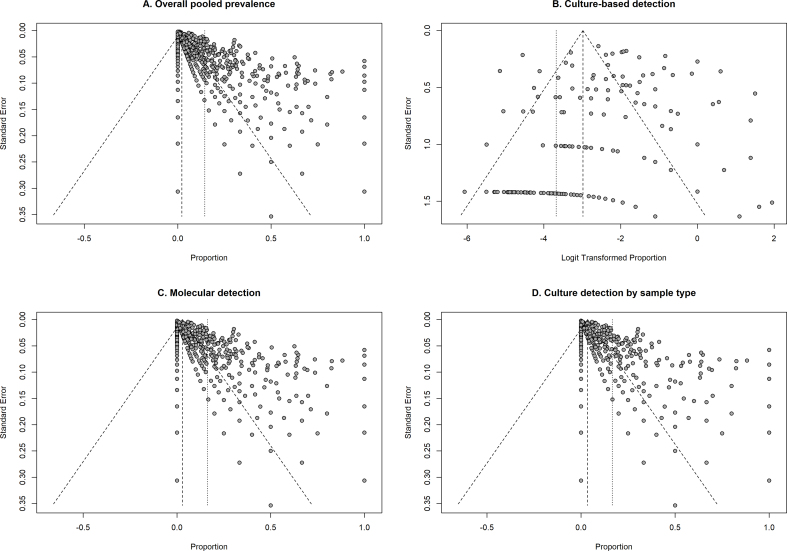

Potential small-study effects and publication bias were explored through visual inspection of funnel plots (Supplementary Fig. 1). Funnel plots were generated for the overall pooled analysis and for the main subgroup analyses, including culture-based detection, molecular detection (Fig. 3), and culture detection according to sample type (Fig. 4). The distribution of studies across the funnel plots showed variability in the dispersion of effect estimates in relation to their standard errors.

Visual inspection may indicate some degree of asymmetry in the distribution of studies, particularly in the overall analysis and in the culture-based detection analyses, where a wider dispersion of estimates was observed among studies with larger standard errors. In contrast, the molecular detection analysis showed a more concentrated distribution of studies around the pooled estimate. Egger's regression test performed for the overall pooled analysis indicated significant statistical asymmetry (*t* = 20.00, *p* < 0.001).

## 4 Discussion

This is the first PROSPERO-registered and validated systematic review providing a comprehensive and integrative overview of leptospirosis in wildlife, encompassing a large diversity of vertebrate taxa investigated in the literature within the selected timeframe. The inclusion of studies that carried out not only isolation, but also other forms of direct detection of *Leptospira*, from a variety of biological samples, has allowed a novel estimate of the frequency of animals presenting infection and urinary shedding of leptospires, depicted by animal taxa.

The meta-analysis revealed that 14.5% of wild fauna worldwide was actively infected with leptospires. This positivity rate is comparable to the 15% reported in a global assessment of *Leptospira* infection in vertebrate hosts [35], despite the markedly different methodological approach adopted in that study, which included studies relying on MAT testing to estimate infection rates.

The proportion of urinary shedding was also estimated, with overall frequency of 16.6%, which is comparable to those reported in domestic species, such as dogs [41] and cats [42], [43], as well as the global seroprevalence found in goats [44], swine [45], and non-human primates [33]. Nevertheless, the estimates found in the present study, both regarding infection and urinary shedding, most likely represent an underestimation of the actual prevalence, given the dynamic nature of leptospiral infection, which varies considerably across time and space. Moreover, most diagnostic methods used for pathogen detection may lack sufficient sensitivity to consistently identify infected hosts. These limitations are primarily attributable to the fluctuating distribution and bacterial load of *Leptospira* within host tissues, blood, and urine, all of which are strongly influenced by the stage of infection, host immunity, infectious dose, and serovar–host adaptation [46]. Some of these methodological constraints were even detected in the subgroup analysis, which showed that direct detection methods based on pooled samples or tissue samples analyzed by IHC yielded markedly higher prevalence rates than molecular detection performed exclusively on urine or kidney samples, thus highlighting that leptospiral infection in wildlife should be addressed using multiple diagnostic approaches and from multiple biological samples. Therefore, the use of pooled tissue samples may increase the likelihood of detecting the bacterium in materials used for culture in future studies, regardless of the stage of infection at the time of sampling.

Despite limited sample sizes for some specific taxa, the results demonstrated a wide range of *Leptospira* prevalence estimates across taxonomic groups, with considerable variation both within and between mammalian and non-mammalian representatives. Didelphidae presented significantly higher positivity for infection and renal shedding, highlighting its importance as carriers of leptospires. This order was represented mostly by specimens of the genus *Didelphis*, which comprises opossums with high ecological flexibility. The extremely generalist diet and wide distribution across several biomes make *Didelphis* among the most frequently encountered synanthropic animals in urban areas in the Americas [47]. These features may be related to the high *Leptospira* infection rates found, as such ecological plasticity leads opossums to explore diverse types of environment and, thus, increase the chances of exposure to leptospires. In addition, synanthropic populations of opossums may show a higher prevalence of *Leptospira* infection due to closer contact with rats, as both animals occupy the same ecological niche in urban settings (*e.g.*, nesting in human dwellings and foraging in domestic garbage) [48]. Indeed, practically all studies describing infection in opossums were carried out in human-wildlife interface landscapes.

Most mammalian orders with sufficiently large sample sizes, and consequently narrower confidence intervals, exhibited infection or renal shedding rates ranging between 10% and 20%, suggesting that mammal species within these taxa may play a significant role in *Leptospira* transmission. Lagomorpha and Afrosoricida, however, showed lower infection rates, suggesting that species within these orders may play a comparatively minor role in local transmission cycles. The reasons for these low prevalences are uncertain, but biases related to the number of studies may have influenced the results: despite the significant number of animals tested, more than 96% of the Lagomorpha specimens were sampled from a single study in France [49], while Afrosoricida individuals were limited to a few insular settings (Réunion Island, Madagascar, and Mayotte).

Our results also indicate significant prevalence among diverse taxa within four orders of reptiles and amphibians. These taxa include animals with very distinct biological and ecological features, such as turtles, snakes, lizards, salamanders, and frogs. Considering this diversity, it is possible that some non-mammalian species may be involved in the epidemiology of leptospirosis - mainly those frequently exposed to water due to semiaquatic habits. However, as isolation from these taxa is rarely described, their contribution to the epidemiology of leptospirosis remains poorly understood. Regarding other non-mammalian animals, the current literature remains extremely scarce.

Prevalence data from all taxa should be interpreted with caution, given that prevalence estimates for infection or urinary shedding may be insufficient to fully capture the ecological significance of different host species in the maintenance and transmission of *Leptospira* [50]. A more comprehensive assessment of their role must also account for population size and structure, geographic distribution, and behavioral ecology, factors that can substantially shape both exposure risk and the likelihood of pathogen dissemination.

In this context, rodents, given their wide distribution, abundance, and high reproductive rates [13], may be of particular importance in sustaining the transmission chain of *Leptospira* in natural settings. With close to 2600 recognized species, Rodentia stands as the largest mammalian order in terms of species richness, accounting for around 40% of all extant mammal diversity [51]. Therefore, the 16.8% prevalence for infection found in Rodentia indicates that wild rodents may play a pivotal role in the epidemiology of leptospirosis. This estimate is even higher than that previously reported by Boey et al. [13], who found that 11.2% of wild rodents, including *R. exulans*, *R. argentiventer*, *R. tanezumi*, and *R. losea*, were infected with *Leptospira* globally. Interestingly, the 24% estimated prevalence found for Cricetidae – the second-largest rodent family, comprising a highly diverse group with hundreds of species distributed worldwide – is comparable to the 24.6% global prevalence reported for *R. rattus* and *R. norvegicus* [13], which are synanthropic rodent species that are considered to be highly exposed to *Leptospira* in urban environments.

Beyond rodents, the study also revealed that bats from the families Nycteridae, Molossidae, and Phyllostomidae exhibited some of the highest prevalence rates. This finding is particularly relevant given that bats account for approximately 25% of all extant mammalian species, with nearly 1400 species distributed worldwide [52]. Their ability to fly, combined with versatile feeding strategies and unique physiological and ecological adaptations that enable colonization of diverse habitats – and their well-documented role as reservoirs for multiple zoonotic pathogens [31] – underscore their potential importance as key players in the dispersion and maintenance of *Leptospira* in natural environments.

Altogether, the evidence derived from multiple meta-analysis models reveals the complexity of wildlife involvement in leptospirosis transmission, yet it remains insufficient to determine the extent to which different species act as maintenance *versus* incidental hosts. Despite the wide range of animals shown to be susceptible to infection, their status as maintenance or incidental hosts for different *Leptospira* strains still needs – in most cases – to be established.

Regarding pathogen diversity and potential serovar-specific associations, our findings demonstrate a wide range of *Leptospira* species circulating among various wild animal hosts across multiple regions worldwide. Similar diversity has been reported in earlier studies, although using different search methods, selection criteria, and study periods. For instance, Hagedoorn et al. [14] provided a meticulous overview of the geographic host distribution and *Leptospira* diversity, yet their review included only studies reporting isolates with serovar-level identification, thereby excluding those based solely on serogrouping or on molecular techniques other than PFGE. These decisions, while methodologically sound within the scope of their objectives, may have inadvertently constrained the inclusion of relevant data on wildlife reservoirs in certain locations, notably those with limited resources to access complex and costly techniques for typing leptospiral isolates at a serovar-level, such as monoclonal antibodies or CAAT. Similarly, other prominent studies, such as the review published by Fratini et al. [26], did not adopt a systematic approach and included serological surveys, making direct comparisons with our results more challenging. Nonetheless, their work provided an important theoretical foundation that contributed to the interpretation of our findings, as well as a valuable source for references included in the snowball procedure, which also included other relevant overview studies.

It is noteworthy that isolation and further characterization were reported in only 104 articles, representing 40% of the studies included, thus limiting the identification of *Leptospira* species in infected animals. Nevertheless, the results indicated that wild rodents harbored the greatest diversity of *Leptospira*, with a total of 14 serogroups detected. Icterohaemorrhagiae was the predominant serogroup isolated from rodents, similar to the long-recognized adaptation of its serovars to synanthropic rodent species. Interestingly, Javanica and Autumnalis were recovered almost exclusively from rodents, possibly revealing host-pathogen specificities. Similarly, Herpestidae and Otariidae appear to be associated with the serogroups Sejroe and Pomona, respectively. The number of isolates, however, was small and derived from only a few geographically restricted studies. Samples from Suidae also showed an apparent association with Pomona, consistent with what has been classically described for swine [53].

The interpretation of serogroup/serovar specificity requires caution, as no reliable inferences should be drawn solely from observational studies. Urinary shedding can be transient and does not necessarily indicate a maintenance status for the host species. To minimize such misinterpretations, future studies should incorporate evidence from mechanistic assays, as emphasized elsewhere [50]. Moreover, since the present analysis used Family and Order as the taxonomic analytical units, future studies adopting a finer resolution at the species level may provide clearer insights into such associations. The availability of the raw data (Supplementary Table 3) may facilitate future studies investigating these evolutionary relationships in greater detail. This approach is particularly relevant given previous evidence suggesting that certain serovars exhibit host specificity, as reported for Pomona in sea lions [54] and Grippotyphosa for raccoons [55], [56].

Molecular characterization of isolates and biological samples revealed the large predominance of *L. interrogans* infecting wildlife fauna, yet *L. kirschneri*, and *L. borgpetersenii* also play a significant role across most animal orders. Notably, however, there is a considerable difference in richness between isolates and direct molecular detections, possibly reflecting the inherent difficulties in obtaining successful cultures, which may preclude proper identification. Additionally, the culture methods employed may be more likely to recover only particular pathogenic species or serovars at the expense of other strains, as has been suggested elsewhere [57], [58].

In the present study, when molecular analysis was combined with serogroup identification, both Chiroptera and Rodentia emerged as potential reservoirs capable of shedding a remarkable diversity of *Leptospira* lineages. For Chiroptera, the inclusion of molecular characterization of biological samples proved particularly valuable, especially given the scarcity of isolates obtained from bats. It remains uncertain, however, whether these findings reflect an inherent plasticity of bats to act as maintenance hosts for a broader diversity of *Leptospira*, or instead simply result from greater exposure of species within these taxa, leading only to transient renal colonization. In any case, the extent of this diversity underscores the considerable potential of bats and rodents to disseminate *Leptospira* into the environment. Still, these results must be interpreted with caution. Rodents and bats together comprise approximately 60% of all mammalian species worldwide [59], and the high *Leptospira* diversity we found among these two orders may reflect their large representation within the global mammalian richness. In this regard, samples from rodents and bats were overrepresented compared with those from the remaining orders. The comparison of different animal taxa in the epidemiology of leptospirosis (as well as other pathogens) has been poorly investigated. Whether bats and rodents are indeed more efficient at carrying multiple pathogens than other taxa, or whether their pathogen diversity simply results from their greater species richness, is still a matter of debate. When comparing both orders, bats can host more zoonotic viruses per species than rodents [60].

Nonetheless, the high species diversity of bats and rodents, combined with specific traits shared by them – including broad geographic distribution, remarkable ecological plasticity that enables colonization of novel niches [29], [32], [61], and perhaps most importantly, the propensity of some species to develop synanthropic behavior – has profound implications for the epidemiology of leptospirosis from a One Health perspective. Rodents and bats may pose a particular threat in human-modified landscapes, where the synanthropic behavior of several species fosters opportunities for cross-species transmission. Some wild rodents are especially adaptable to disturbed environments and are frequently recorded in urban and peri-urban areas, including species of *Peromyscus* [62], *Oligoryzomys* [63], *Calomys* [64], *Mastomys*, and *Arvicanthis* [65]. Likewise, several bat taxa are found in similar scenarios, including some genera that are highly adapted to urban areas, like *Molossus*, *Eumops*, *Myotis*, and *Rhinolophus* [66], [67].

As ongoing land-use changes and deforestation, compounded by global warming and its recognized influence on infectious disease dynamics [68], are expected to intensify interactions at the human–wildlife interface, it is reasonable to consider that the frequency of *Leptospira* transmission mediated by wild reservoirs, notably Rodentia and Chiroptera, is likely to increase in the coming decades [69], [70], as has been documented for other zoonotic pathogens [71], [72].

This is particularly relevant considering that the total biomass of terrestrial wild mammals is estimated to reach approximately 20 million tonnes [73], of which 23% are represented by Rodentia and Chiroptera combined. Although this biomass is still considerably smaller than that of domesticated mammals, which currently outweigh all wild mammals by roughly tenfold [73], [74], the epidemiological relevance of wildlife as a potential source of environmental contamination should not be overlooked. Livestock are often raised under more controlled management conditions, which may reduce opportunities for pathogen inter-species transmission. In contrast, wildlife populations occur across a wide range of natural and peri-urban environments and may therefore contribute to the environmental dissemination of *Leptospira*. This perspective becomes even more relevant when considering that reservoir hosts may shed leptospires in urine continuously for prolonged periods, with median shedding quantities ranging from 10^5^ to 10^8^ cells per day [75], potentially exerting a profound effect on environmental pathogen pressure [76]. Additionally, as human–wildlife interfaces are often concentrated in areas of irregular and uncontrolled land occupation, socially and economically marginalized human populations may be at higher risk [77]. Illegal trafficking, bushmeat consumption, and the trade of wildlife-derived medicinal products may exacerbate even more pathogen transmission from wildlife within these communities [78], [79].

Altogether, our findings underscore the urgent need for integrative strategies that connect public health, environmental management, and wildlife conservation. Within this framework, both passive and targeted surveillance of pathogens circulating among wildlife, domestic animals, synanthropic species, and humans emerge as central components of the One Health paradigm. Yet, the development of novel methodologies aimed at improving *Leptospira* isolation remains a cornerstone for advancing our understanding of leptospirosis ecology. At the same time, molecular and bioinformatics approaches capable of inferring serovar-level classification directly from genetic data, particularly those targeting *rfb* cluster genomic regions, which apparently show a strong correlation with *Leptospira* serovar designation [22], represent promising alternatives that can substantially broaden the scope of surveillance and facilitate serovar prediction without the need for culture. Equally important, researchers should be encouraged to openly share their isolation outcomes and genomic datasets in public repositories, such as the platform established and maintained by Hagedoorn et al. [14], to promote data integration and global collaboration in leptospiral research.

This study presents several limitations. First, the literature search was restricted only to recent publications, thereby precluding comparisons with older data. This decision was taken to capture the most up-to-date epidemiological profile of leptospirosis and to include studies employing more advanced diagnostic and typing techniques, within a period marked by continuous improvements in *Leptospira* taxonomy and classification. Moreover, no spatial-temporal analysis was conducted, which limited our ability to detect potential changes in epidemiological patterns over time. Studies relying solely on the MAT were also excluded. This methodological decision was based on the inherent limitations of this technique, which, as an indirect serological test, may bias acute infection frequency estimates. Furthermore, MAT results show poor correlation with renal shedding of leptospires and weak association with the infecting strain, as extensively documented in the literature [6].

Several studies reporting leptospiral isolation from wild animals were not included in the analysis of infecting strains. Their exclusion was justified by the lack of sufficient information regarding the host species from which samples were collected, the methods employed for strain characterization, or both. In some cases, data were reported in an aggregated manner, making it impossible to ascertain host-specific characteristics.

Although studies conducted in zoos, rehabilitation centers, under management conditions, or rescue facilities were identified, no subgroup analysis was performed to evaluate the influence of captivity on positivity rates or on the diversity of host species and serogroups detected. This omission is acknowledged as a limitation, since artificial conditions in such environments may induce behavioral and dietary alterations, elevate stress levels and susceptibility to infection, and facilitate cross-transmission from synanthropic or domestic animals [37], [80]. These managed conditions may include wildlife species maintained in farm systems, which in some regions represent semi-managed populations rather than conventional domestic livestock. These factors could produce epidemiological patterns that diverge from those observed in free-ranging populations. Nevertheless, only 18 studies were conducted exclusively on captive or managed animals.

Alongside these limitations, the risk of bias assessment revealed several limitations across the included studies, frequently arising from insufficient description of the criteria used to define studied populations. It is important to note, however, that many of these limitations reflect the purposes of the present review rather than shortcomings of the original investigations on wildlife infection. Common issues included the lack of species-level reporting and the failure to distinguish results according to animal origin or sampling site. In addition, several studies relied exclusively on PCR without sequencing to determine infection status, preventing verification of potential false positives. A further limitation was that many studies reported data only in aggregated form, hindering more detailed evaluations. Furthermore, funnel plot asymmetry observed in the analyses should be interpreted with caution. In prevalence meta-analyses involving diverse wildlife hosts, diagnostic methods, and sampling strategies, such asymmetry may reflect underlying biological and methodological heterogeneity rather than true publication bias. Future efforts addressing leptospirosis in wildlife should make raw data available as supplementary material, thereby enabling reanalysis and fostering greater transparency for both researchers and readers.

## 5 Conclusion

Our findings demonstrate that wildlife plays a crucial role in the maintenance and dissemination of *Leptospira* within natural ecosystems. By synthesizing two decades of studies reporting direct detection of *Leptospira spp.* in wild animals worldwide, this systematic review and meta-analysis provides comprehensive evidence of the pathogen's global ecological scope. As human-driven environmental changes continue to reshape landscapes and intensify contact at the human–wildlife interface, the risk of leptospiral spillover is likely to escalate.

We hope that our findings reinforce the need for integrated surveillance frameworks grounded in a One Health approach, recognizing that the dynamics of leptospirosis emerge from the complex interactions among wildlife, domestic animals, humans, and their shared environments. Advancing the understanding and control of leptospirosis will therefore depend on interdisciplinary efforts that bridge ecological, veterinary, and public health perspectives. In this context, improving *Leptospira* isolation techniques and expanding the use of innovative molecular approaches will be essential to better elucidate the role of wildlife species in the epidemiology of the disease.

The following are the supplementary data related to this article.

Supplementary Table 1

Supplementary Table 2

Supplementary Table 3

Supplementary Table 4

Supplementary Table 5

Supplementary Table 6

## CRediT authorship contribution statement

**Stephanie Bergmann Esteves:** Writing – review & editing, Writing – original draft, Validation, Software, Project administration, Methodology, Investigation, Formal analysis, Data curation, Conceptualization. **Ana Carolina Monteiro Miranda Grolla:** Writing – review & editing, Formal analysis, Data curation. **Adriana Cortez:** Writing – review & editing, Formal analysis, Data curation. **Juliana de Paula Nhanharelli:** Writing – review & editing, Writing – original draft. **Evelyn Moura de Lima:** Writing – review & editing, Formal analysis, Data curation. **Felipe Fornazari:** Writing – review & editing, Writing – original draft. **Luiz Gustavo Melo da Silva:** Writing – review & editing, Formal analysis, Data curation. **Mariana Vitória Ramos do Amaral:** Writing – review & editing, Writing – original draft. **Bruno Alonso Miotto:** Writing – review & editing, Writing – original draft, Visualization, Supervision, Project administration, Methodology, Formal analysis, Data curation, Conceptualization.

## Declaration of competing interest

The authors declare that they have no known competing financial interests or personal relationships that could have appeared to influence the work reported in this paper.

## Data Availability

Data will be made available on request.
